# Influence of body size, topography, food availability and tree-fall gaps on space use by yellow-footed tortoises (*Chelonoidis denticulatus*) in Central Amazonia

**DOI:** 10.1371/journal.pone.0211869

**Published:** 2019-02-22

**Authors:** Aline S. Tavares, Thais Queiroz Morcatty, Jansen Zuanon, William E. Magnusson

**Affiliations:** 1 Programa de Pós-Graduação em Ecologia, Instituto Nacional de Pesquisas da Amazônia, Manaus, AM, Brazil; 2 Oxford Wildlife Trade Research Group, Department of Social Sciences, Oxford Brookes University, Oxford, United Kingdom; 3 Coordenação de Biodiversidade, Instituto Nacional de Pesquisas da Amazônia, Manaus, AM, Brazil; College of Agricultural Sciences, UNITED STATES

## Abstract

Habitat selection and extension of the area used by a given species may vary during different phases of its life and are often determined by the distribution and availability of resources throughout the landscape, such as food, breeding sites, and shelters. In this study, we assessed the influence of body size on the areas used by 21 individuals of the yellow-footed tortoises (*Chelonoidis denticulatus*) from January to June 2017 in a dense rain forest area in Central Amazonia. We also investigated whether individuals selected different ranges of terrain slope, elevation, areas with high food availability, or areas with treefall gaps that could be used for shelter or thermoregulation. We monitored tortoise movements using thread-bobbins, and sampled terrain characteristics, availability of potential food resources and forest gaps along the routes used by the tortoises. We also measured the same variables in plots distributed systematically throughout the study area to evaluate resource availability. Tortoises used an average area of 1.56 ha (SD = 1.51, min = 0.03, max = 6.44). The size of the area used was positively associated with the individual body size, but did not vary between sexes. Small individuals selected higher and flatter areas where the availability of fallen flowers was higher, whereas the area used by larger individuals did not differ from the natural availability of topographic traits and food in the region. Although tortoises did not select areas according to availability of tree-fall gaps, most larger tortoises were found sheltered under fallen trees (85%). Conversely, small individuals were mainly found hidden under litter (66%). Body size determined the patterns of landscape use by tortoises; larger individuals were mainly generalists, but small individuals occupied high and flat areas. The yellow-footed tortoise is endangered by hunting, illegal collection for the pet trade, habitat destruction and effects of climate change. Size-related differences in habitat selection should be taken into account in species-distribution models for the identification of suitable areas for reintroduction and the development of management plans in protected areas.

## Introduction

Space use by animals is affected by physiology and behavior, and these often vary among individuals of different sexes and body sizes [1]. The distribution of food resources [2] and the availability of breeding sites often constrain the individual’s movement patterns. However, for ectothermic animals, such as terrestrial chelonians, the presence of thermoregulation sites and shelters, which enable them to be active when necessary, and to maintain minimal metabolic rates and low predation risk at other times [1], may be more important.

Topography is a key driver of environmental heterogeneity and consequently affects the spatial distribution of resources. Several studies in dense rain forest in Central Amazonia have detected the influence of slight topographical variation on animals, even where the elevation range does not exceed 100 meters. These studies have associated the species distributions of ants [3], bats [4], birds [5] and anurans [6,7] to resource distribution along the topographic gradient. However, the influence of variation in slope and elevation on intraspecific behavior has not been studied. The distribution of resources along topographic gradients may also lead to differential space use by individuals of different sexes and body sizes.

Yellow-footed tortoises (*Chelonoidis denticulatus* Linnaeus, 1766) are widely distributed in the Amazon. Studies of *C*. *denticulatus* [8] (in other regions of Amazonia) have indicated habitat selection by the tortoises [9,10]. Soil type [11], vegetation composition [12,13,14,15] as well as tree biomass [16] and tree falls [17] vary along the topographic gradient in this region. Therefore, the topographic gradient may determine the distribution of nesting sites, food sources, such as fruit, flowers and fungi [9], and the presence of tree fall gaps, which are often used by the yellow-footed tortoises for shelter and thermoregulation sites [8,9,18].

Although the distribution of *C*. *denticulatus* covers the entire Amazon Basin [19], the use of space by the species has been studied only on the biome borders [1,18,9,10,20]. It is likely that diet and habitat use vary with tortoise size but, space use by small yellow-footed tortoises has not been studied due to the low detectability of smaller individuals in the forest.

Conservation actions for *C*. *denticulatus* have been developed in several parts of its distribution due to overhunting [21,22,23,24,25] and recent studies have suggested the reintroduction of *C*. *denticulatus* individuals to restore ecological processes, especially seed dispersal, in habitats that have been subject to the extinction of large frugivores [26]. For the development of management plans or the selection of reintroduction areas, information on local suitability and habitat selection by *C*. *denticulatus* of all sizes is required.

In this study, we investigated space use by *C*. *denticulatus* in a dense rainforest in the Central Brazilian Amazon and evaluated the influence of topography and distribution of potentially-important resources on habitat selection by the species. We aimed to answer the following questions: i) Does the extent of area used vary according to sex and body size of individuals? ii) Do individuals select restricted slope and elevation ranges? iii) Do individuals select areas with higher food availability and/or more forest gaps?

## Materials and methods

### Study area

We conducted fieldwork in Adolpho Ducke Forest Reserve (Ducke Reserve hereafter), a 10-thousand ha protected area located on the outskirts of Manaus, Amazonas, Brazil (2°56'50"S, 59°55'49"W at the reserve headquarters). The reserve is covered by dense *terra firme* rainforest over acidic nutrient-poor soils [11] and an undulating topography. Clayey oxisols predominate in the higher areas and are gradually replaced by sandy soils in the lower areas [11]. The dry season generally occurs between July and September, but only two months, on average, have precipitation below 100 mm [27]. The mean precipitation, temperature and relative humidity between January and June of 2017, were 284 mm, 26°C and 95%, respectively (data obtained from a weather station in Ducke Reserve). A grid system of trails following the RAPELD model was installed in 2001 [28]. Data collection was conducted along trails located on the edge region of the grid system. The study site (4,500 ha) is crossed by four 8 km trails, which give access to 32 systematically distributed 250 m long permanent plots.

### Capture and monitoring of tortoises

From January to June 2017, we used two methods to capture yellow-footed tortoises. The first method was active searching an area within approximately 20 m of the trails from 7:00 to 18:00 We gave special attention to areas around fallen trees, branches and holes. Active searching was complemented by the use of baited pitfall trap (0.7 m deep x 1 m diameter) distributed over the study area (> 1 km distant from each other). We used 32 traps that were active for 10 consecutive days, and suspended baits (rotten meat, chicken or fish) were used to attract tortoises.

We determined sex by using a combination of external morphological traits [29] and measured the maximum straight-line length of the carapace, given by the linear distance between the anterior and posterior carapace extremities. Individuals < 25 cm were considered as “small” and ≥ 25 cm as “large” [30]. Due to the lack of accuracy in identifying external sexual morphological traits, we classified small individuals as “unknown sex”. The captures and data collection were carried out following ethical and legal procedures required by the Instituto Chico Mendes de Conservação da Biodiversidade (SISBIO n°. 56715–1) and were approved by the Ethics Committee of the National Institute for Amazonian Research (n°.052/2016).

We recorded the exact location where each individual was captured with a GPS (Garmin 76CSX). To track individual movements, we attached thread-bobbins to the carapace. This method is cheaper than radiotelemetry and allows collection of fine details on individual routes. To avoid loss of data due to the line breaking, for the highly-dispersive larger tortoises we used two reels simultaneously, one with a thin 1,000 m thread (100% polyester) and the other with a more resistant 100% cotton (n° 10) line that was 440 m long. For small individuals, we used only the thinner line. Thread-bobbins did not exceed 5% of individual’s body mass and, when necessary, we changed the reels with minimal manipulation to avoid interfering with the individual’s displacement.

We attempted to monitor individual tortoises for as long as possible, but due to line breakages tortoises were monitored for periods ranging from 3 to 31 days (Table 1). Whenever possible, we located all the individuals each day at different times of day. When sighted, we recorded the current behavior (walking, eating or sheltered), and the type of cover for sheltered tortoises. When we could not find an individual on a given day, we calculated the average daily displacement through the total route monitored. Although not very accurate, we used this value to roughly estimate the distance moved that day, assuming that the low frequency of such events did not bias our estimatives of mean and total distance covered by the individuals.

**Table 1:** Sex, size, movement, and behavior of *Chelonoidis denticulatus* captured in Adolpho Ducke Forest Reserve, Central Amazonia.

Individual	Sex	Body length * (cm)	Used area (ha)	Sampling days	Behavior			Shelter type		
					Sheltered	Walking	Feeding	Fallen trees	Litter	Palm roots
1^a^	Male	26.1	-	-	0	1	0	-	-	-
2 ^a^	Female	31.8	-	-	0	1	0	-	-	-
3 ^a^	Female	33.6	-	-	0	1	0	-	-	-
4 ^a^	Unknown	13.3	-	-	0	1	0	-	-	-
5	Unknown	18.4	0.37	3	0	1	0	-	-	-
6	Male	39.7	1.66	5	0	1	0	-	-	-
7	Unknown	10.7	0.15	30	4	2	0	1	2	1
8 ^a^	Unknown	9.0	-	-	0	1	0	-	-	-
9	Female	32.2	2.45	4	5	0	0	5	0	0
10	Male	34.4	1.87	4	4	1	0	2	2	0
11	Female	28.2	2.33	5	5	0	0	3	2	0
12	Female	37.9	2.04	15	5	2	1	5	0	0
13	Male	30.9	6.44	29	14	1	0	12	2	0
14	Male	31.0	1.09	4	0	1	0	-	-	-
15	Male	25.3	1	31	6	1	1	5	1	0
16	Unknown	21.1	1.51	5	1	0	0	0	1	1
17	Male	36.0	1.97	4	-	-	-	-	-	-
18	Unknown	23.1	0.68	4	1	1	0	0	0	1
19	Unknown	5.5	0.03	14	5	4	0	0	5	0
20	Unknown	20.6	1.23	6	4	0	2	2	2	0
21	Female	30.3	0.23	4	4	0	0	4	0	0

* Maximum straight-line carapace length

^a^ Individuals not monitored.

### Area used

We divided the individual routes taken by the tortoises in 10 m segments. The location of each segment was recorded with a GPS and the direction to the next point was measured in degrees with a compass. Terrain slope was measured with a clinometer.

To estimate the area covered by the tortoises during the monitoring period, we used a minimum convex polygon (MCP) that included all the 10 m points along the route. Polygons were constructed using both GPS and compass data, but we present only the results based on GPS because they were highly correlated (Pearson correlation coefficient: r = 0.98, n = 8).

### Environmental variables

We obtained 30 m resolution terrain-elevation data from SRTM-HASL (Shuttle Radar Topography Mission—Height Above Sea Level), available at http://earthexplorer.usgs.gov/. We estimated the terrain slope by calculating the altitudinal difference between adjacent pixels according to the *Slope* function in Arc Map 10.2 software and calibrated them by using our on-the-ground records (Pearson correlation coefficient: r = 0.64, n = 856). To estimate the availability of the slope and elevation values in the study area, we randomly selected 2,000 points using the *random Points* function in R software and extracted the values for each variable. We calculeted the median slopes and elevations used by individual tortoises. To test the relationships between slope and elevation and other environmental variables, we used the mean value for each 250 m center line of the permanent plots in the study area.

Availability of fresh fruits, flowers and fungi in the study area was evaluated in four periods between January and June 2017. We searched for food resources close to the ground (up to 20 cm high) along a 0.5 m wide strip that followed the center lines of the permanent plots (total area = 4,000 m^2^). We used the mean values measured in g/m^2^ per plot in the food-availability analyses. Simultaneously, we estimated the density of the same food sources along the routes used by tortoises. We collected data in a 20 cm wide strip along the routes. Food items encountered were weighed on a precision digital scale.

We took canopy photographs along the center line of 29 of the 250 m long permanent plots to estimate the proportion of the forest covered by tree-fall gaps, and each 10m along the tortoise routes to estimate the proportion of routes covered by tree-fall gaps. Photographs were taken with a Canon A3400 IS camera positioned one meter above ground and we visually categorized the photographs into "gap" or "non-gap". The relative frequency of gaps in plots and along tortoise routes was used to infer selection by tortoises.

### Statistical analyses

We used simple linear regressions to test if the density of food resources and the relative frequency of forest gaps were related to different degrees of the terrain slope and elevation. In order to meet the assumption of normality, we log_10_ transformed the food-resource data.

We calculate the relative frequency of each behavioral class based on the proportion of encounters. We excluded individuals with < 3 encounters to calculate the relative frequency of use of each type of shelter. Shelter-use by small and large individuals was analyzed separately.

We used Generalized Linear Models (GLM) to assess the influence of sex and body length on the area used. We included the number of days the individual was monitored in these models to remove the effects of differences in sampling effort. We tested across a wide set of distribution families and selected the Log Normal family as the best-fit.

The Akaike Information Criteria (AIC) was used to select the distribution of families and final models. We considered models with ΔAIC < 2 equally adjusted and selected the model with fewer variables [31].

To investigate whether individuals used terrain slope and elevation according to their availability in the study area, we compared the 2000 random values within the study area to the values used by the tortoises along their paths using the Kolmogorov-Smirnov test. In order to test if the use of different slopes and elevations by the tortoises was affected by their body length, we used simple linear regressions between the medians of each variable for each individual. We also tested whether these variables affected the variability (standard deviation) in the use of slopes and elevations by the tortoises.

In order to test whether tortoises selected areas with more food resources (flowers, fungi and fruit) or forest gaps, we compared the medians of data from individual routes to those of the plots with the Mann-Whitney test. To test the effect of body length on the selection of areas with greater availability of food resources, forest gaps, and fallen trees, we used simple linear regressions. Analyses were undertaken in R software (http://www.R-project.org/) with the *gamlss*, *vegan*, *sp*, *raster* and *dismo* packages. We considered p≤0.05 to be statistically significant.

## Results

### Environmental characteristics

**Fig 1:**
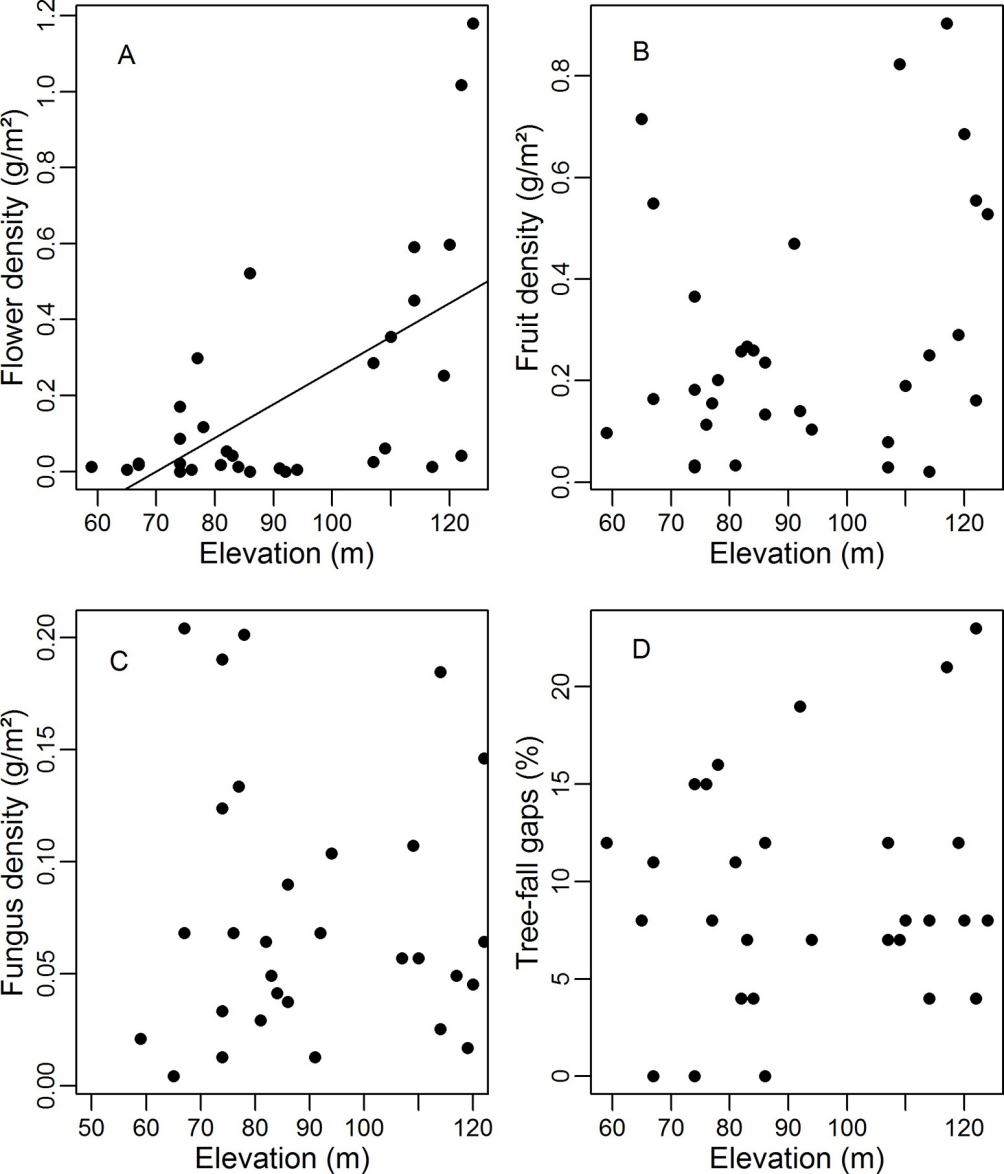
**Flower (A), fruit (B) and fungus (C) densities, and tree-fall gap percentages (D) per plot in relation to terrain elevation**. Regression line given only for the statistically-significant (p ≤ 0.05) model.

**Fig 2:**
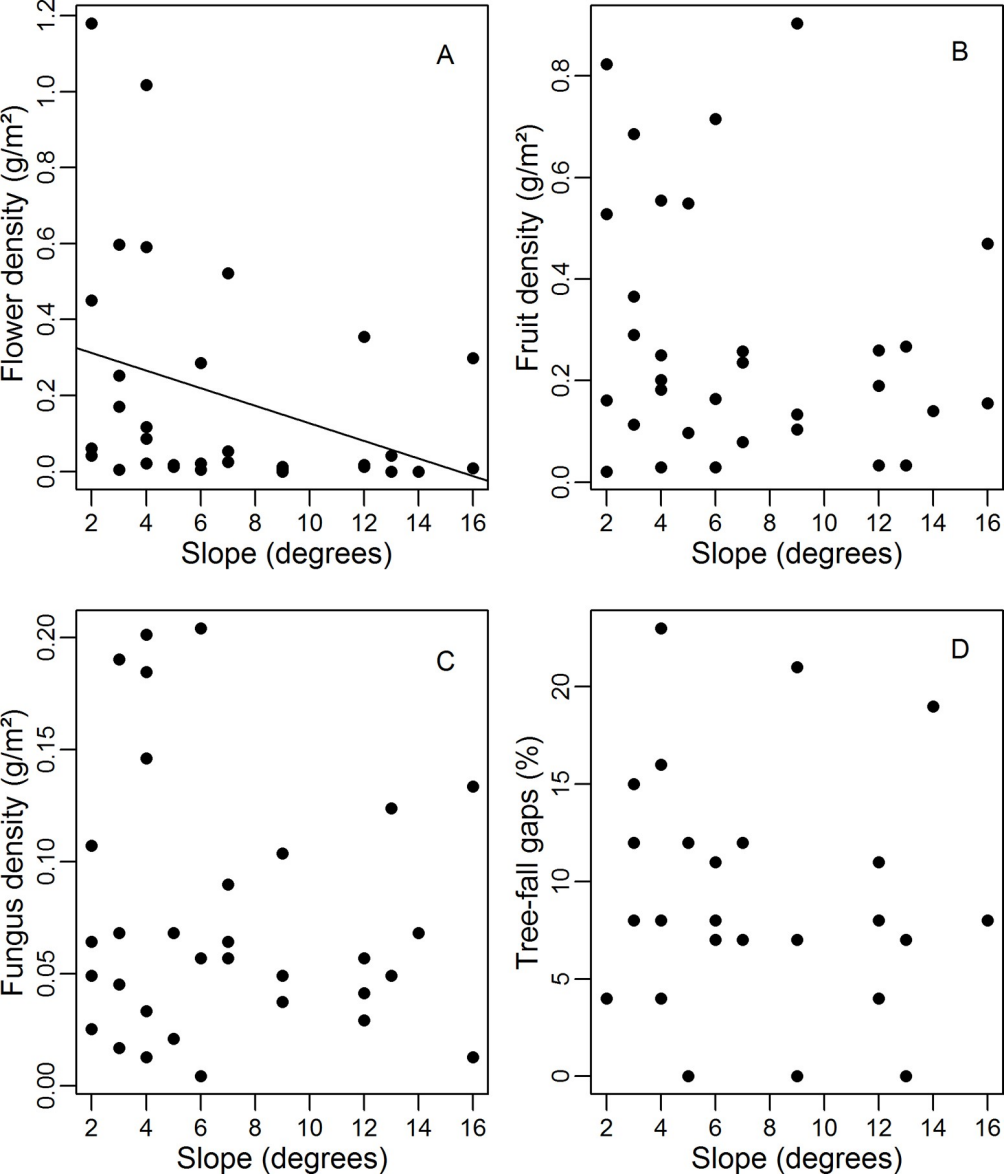
**Flower (A), fruit (B) and fungus (C) densities, and tree-fall gaps percentages (D) per plot in relation to terrain slope**. Regression line given only for the statistically-significant (p ≤ 0.05) model.

The modal terrain elevation in the area was 120 m (mean = 92, SD = 20.2, min = 28 and max = 127) and modal terrain slope mode was 2° (mean = 7, SD = 4.3, min = 0 and max = 25). Flower density was greater in higher elevation areas (Simple linear regression: F_1,30_ = 16.07, r^2^_adj_ = 0.32, p < 0.01; Fig 1A) and lower slope (F_1,30_ = 3.82, r^2^_adj_ = 0.08, p = 0.05; Fig 2A). However, fruit and fungus densities (Fig 2B and 2C) did not differ significantly between areas with different degrees of slope (Simple linear regression: fruits: F_1,30_ = 1.10, r^2^_adj_ = 0.003, p = 0.30; fungus: F_1,30_ = 0.35, r^2^_adj_ = -0.02, p = 0.55) or elevation (Fig 1B and 1C) (Simple linear regression: fruits: F_1,30_ = 2.66, r^2^_adj_ = 0.05, p = 0.11, fungus: F_1,30_ = 0.16, r^2^_adj_ = -0.02, p = 0.68). There was no significant relationship between the presence of tree-fall gaps and slope (Simple linear regression: F_1,27_ = 0.20, r^2^_adj_ = -0.02, p = 0.65; Fig 2D) or terrain elevation (Simple linear regression: F_1,27_ = 0.61, r^2^_adj_ = -0.01, p = 0.43; Fig 1D). Of the sampled food resources, only fungus density varied during the sampling period (ANOVA: fungus: p = 0.05, fruits p = 0.17, flowers: p = 0.29).

### Space use by *Chelonoidis denticulatus*

We captured 21 yellow-footed tortoises (8 small individuals of unidentified sex, 7 males and 6 females) and were able to monitor the movements of 16 (6 small, 6 males and 4 females). We captured 18 individuals by active search and only three individuals in pitfall traps. In 71% of encounters (n = 58) individuals were found in shelters, 24% were walking, and 5% were feeding. In 85% of encounters with sheltered larger tortoises, the individuals were using fallen trees and 15% were in litter. However,66% small individuals were in litter, 25% were under tree-falls and 8% beneath palm roots (Table 1).

**Fig 3:**
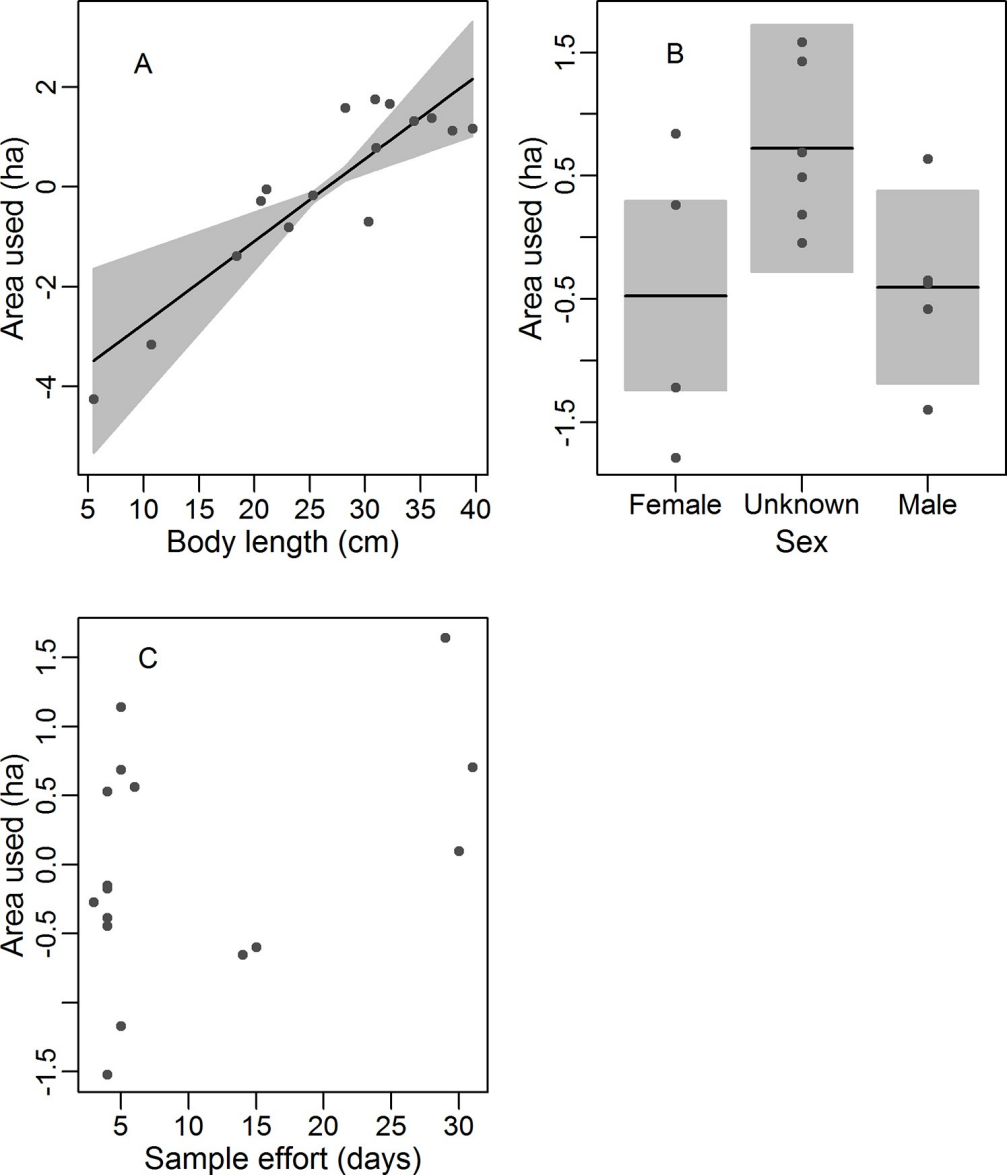
**Partial regressions derived from a generalized linear analysis of covariance model of the effects of (A) the body length, (B) sex, and (C) sampling effort on area used (ha)**. The y-axis values are simple regression partials, and x-axis values are given as partial values added to mean variable values. The grey areas span the 95% confidence intervals. The regression line in (A) represents the significant model (p ≤ 0.05). Sex was a categorical variable and the black lines in B represent category means.

We recorded an average area of 1.56 ha used by the monitored tortoises (SD = 1.51, min = 0.03, max = 6.44) (Table 1). The areas used were larger for larger individuals (GLM: Estimate: 0.16, SE = 0.04, t = 3.77, p < 0.01, Fig 3A) and did not vary by sex (GLM: female = 0.07, SE = 0.50, t = 0.13, p = 0.89, Fig 3B) or sampling effort (GLM: Estimate = 0.03, SE = 0.02, t = 1.4, p = 0.18, Fig 3A).

**Fig 4:**
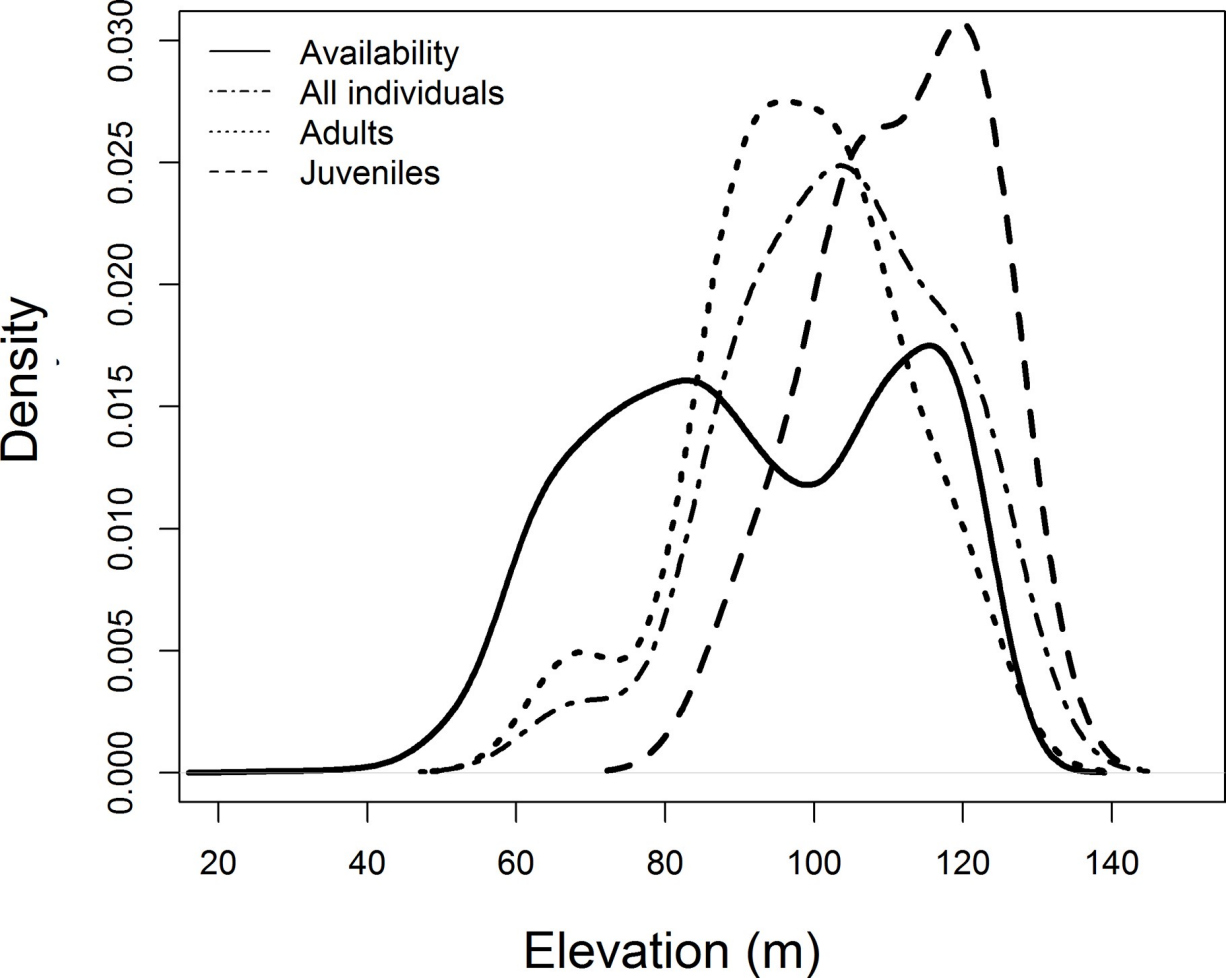
Distribution of elevations available and used by yellow-footed tortoises (n = 21).

**Fig 5:**
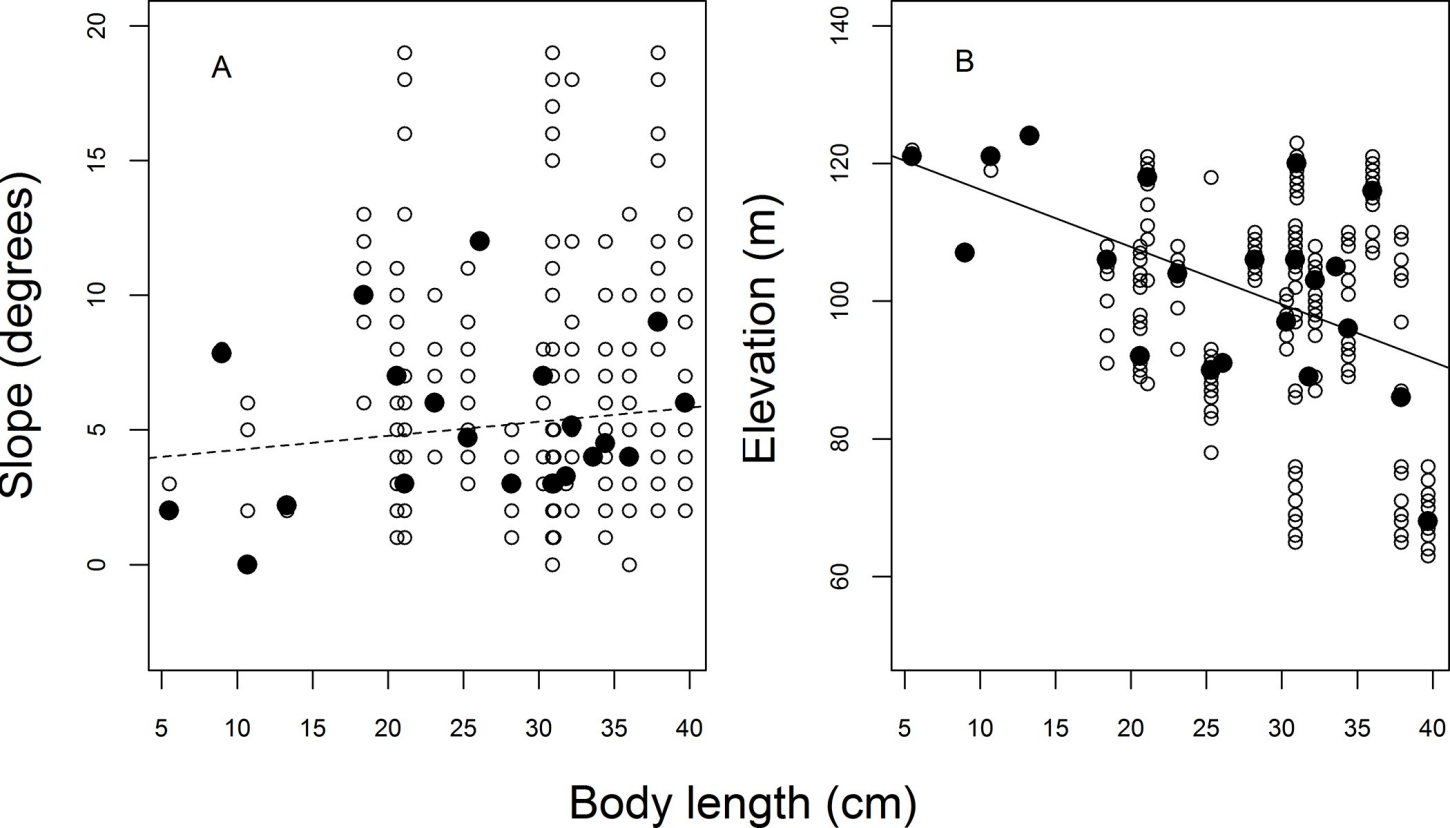
**Relationship between body length and slope (A), and elevation (B) used by yellow-footed tortoises.** The trend lines represent the regression between the medians of the elevation and slope values used by the yellow-footed tortoises and their respective body length. The continuous regression line represents the statistically significant model (p ≤ 0.05).

**Fig 6:**
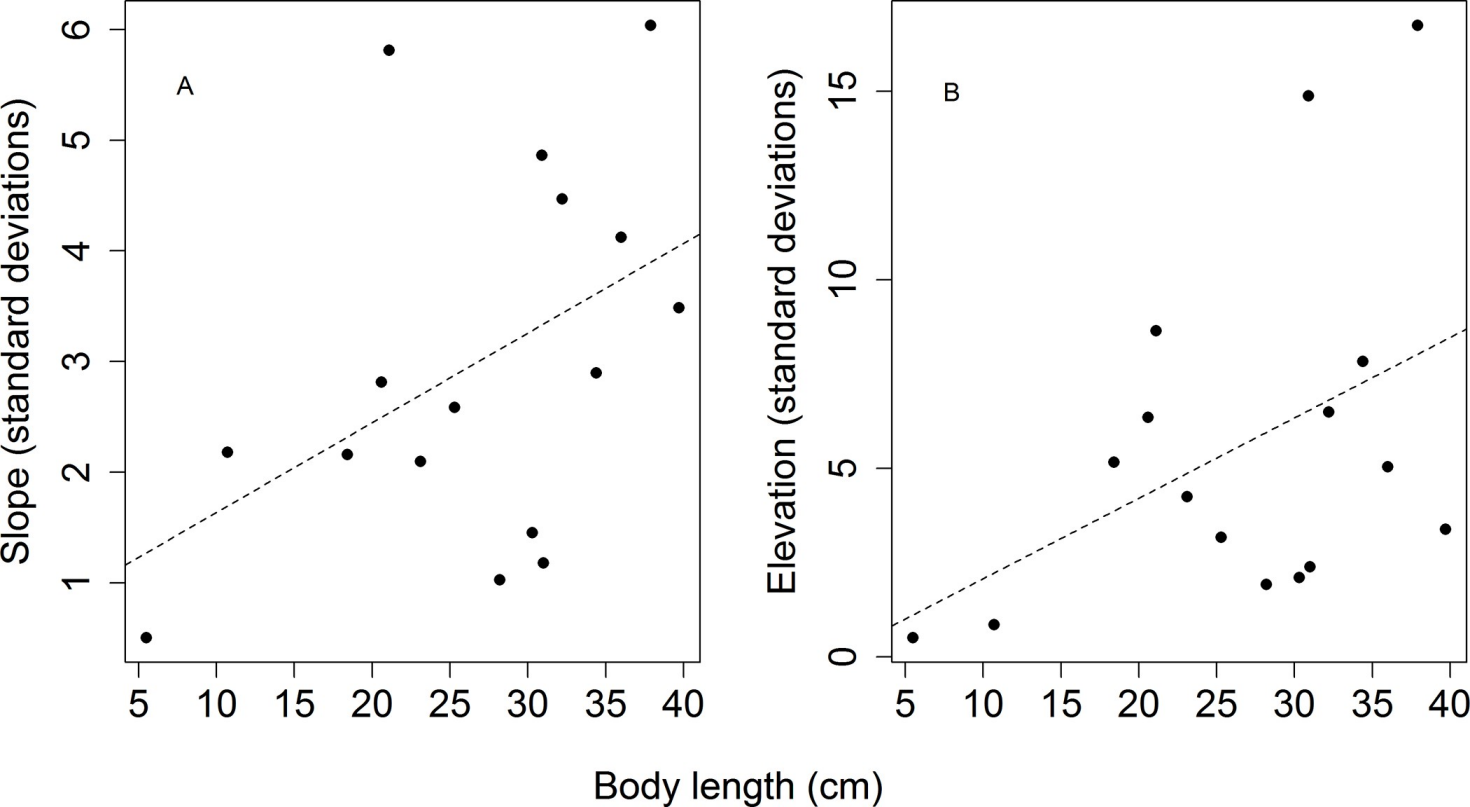
**Relationship between body length and variation (standard deviation) in the use of terrain slope (A), and elevation (B) by the yellow-footed tortoises.** The dotted lines represent statistically models that were statistically non-significant, but with p < 0.08 in all cases.

The terrain slopes used by all tortoises taken together (Fig 4) did not differ significantly from those available in the study area (Komogorov-Smirnov test: D = 0.21, p = 0.27) and there was no statistically-significant relationship between individual size and terrain slope used (Simple linear regression: F_1,19_ = 0.59, r^2^_adj_ = -0.02, p = 0.45), but individuals smaller than 15 cm (n = 4) were not recorded in areas with slopes higher than 8 degrees (Fig 5). The yellow-footed tortoises used higher areas more frequently than would be expected based on their availability in study area (Komogorov-Smirnov test: D = 0.37, p = 0.005). This relationship (Fig 4) was stronger for small individuals (body length < 25cm) (Komogorov-Smirnov test: D = 0.52, p = 0.02) than for large individuals (body length ≥ 25cm) (Komogorov-Smirnov test: D = 0.34, p = 0.09). Individuals with greater body length used lower areas more than smaller individuals (Simple linear regression: F_1,19_ = 9.40, r^2^_adj_ = 0.29, p < 0.01; Fig 5). The standard deviations of the elevation and slope of areas used by tortoises (Fig 6) were larger for larger individuals, indicating that these individuals use a greater range of slopes and elevations, while smaller individuals tended to remain in higher and flat areas (Simple linear regression: elevation: F_1,14_ = 3.36, r^2^_adj_ = 0.13, p = 0.08, slope: F_1,14_ = 3.78, r^2^_adj_ = 0.15, p = 0.07).

**Fig 7:**
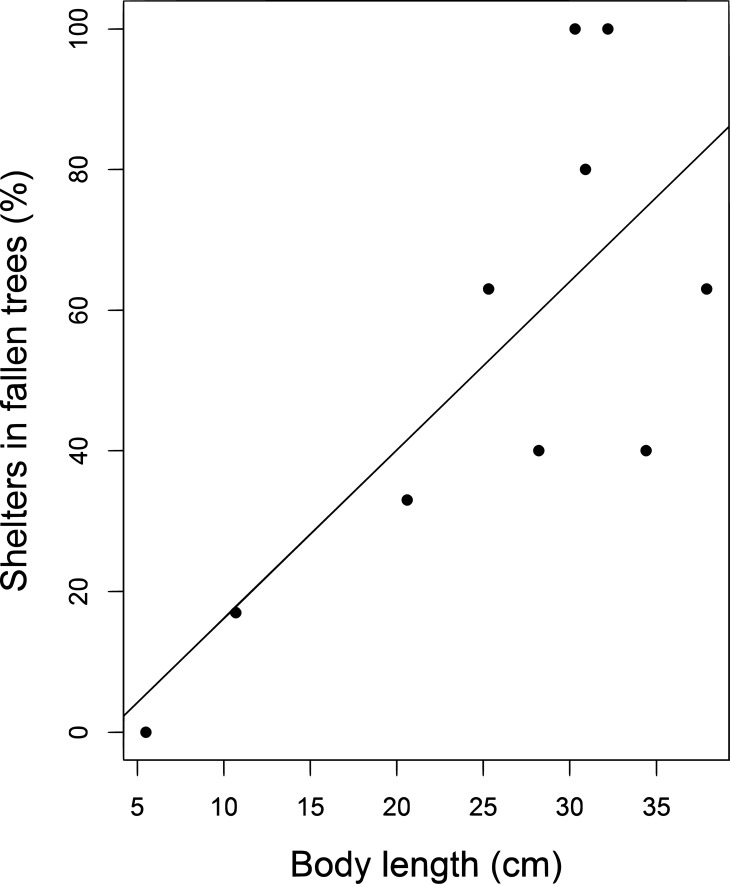
Relationship between the percentage of encounters under fallen trees and individual body length.

The relative frequency of forest gaps along the tortoise routes was not significantly different from that available in the area (Mann Whitney test: W = 218; p = 0.74) and also did not vary in relation to individual size (Simple linear regression: F_1,14_ = 1.77, r^2^_adj_ = 0.04, p = 0.20). However, larger individuals used more fallen trees as shelter than did smaller individuals (Simple linear regression: F_1,8_ = 9.8, r^2^_adj_ = 0.49, p = 0.01; Fig 7).

**Fig 8:**
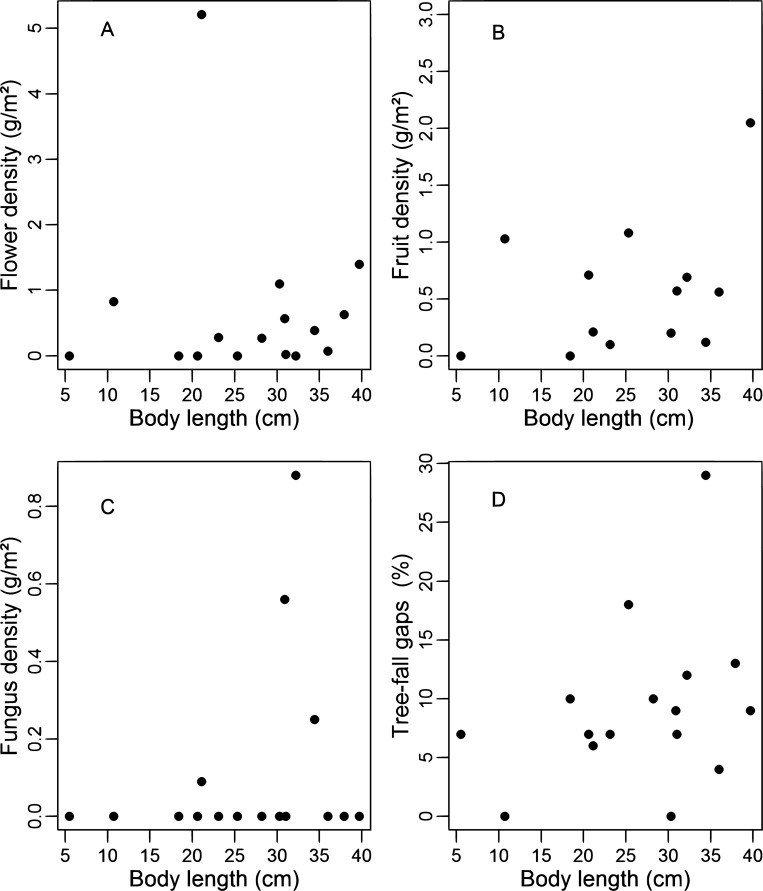
Flower (A), fruit (B) and fungus (C) densities, and relative frequency of tree-fall gaps along routes taken by tortoises (D) in relation to body length.

Flower and fruit densities (g/m^2^) available along the tortoise routes were similar to the availability of those resources in the area (Mann-Whitney test: flowers: W = 238, p = 0.70; fruits: W = 251, p = 0.91), but fungus density was lower along the tortoise routes than generally available in the study area (Mann-Whitney test: W = 94; fungus: p < 0.01). The density of other food resources in the areas used by tortoises (Fig 8) was not significantly related to body length (Simple linear regression—flowers: F_1,14_ = 0.03, r^2^_adj_ = -0.06, p = 0.86; fruits: F_1,14_ = 1.28, r^2^_adj_ = 0.01, p = 0.27; fungus: F_1,14_ = 0.69, r^2^_adj_ = -0.02, p = 0.41).

## Discussion

Small variations in terrain slope and elevation may influence the distribution of soil characteristics [11], plant assemblages [13,15] and several animal species [3,6,5,4] in Central Amazonia. According to our findings, these variations also influence the use of space by *C*. *denticulatus* individuals in Ducke Reserve, especially by smaller individuals.

The areas estimated to be used by the tortoises (0.03 to 6.44 ha) were considerably smaller than estimates from previous studies on the same species, which varied from 23 to 66.5 ha (n = 6) in the Northern Amazonia [1], around 101.49 ha in Southeastern Amazonia (n = 22) [29], and from 9.7 to 332 ha (n = 8) in Southwestern Amazonia [10]. However, there was wide variation in the used area within and among these studies, all of which were carried out in ecotones and mosaics composed by different vegetation types, such as savanna and mountain forest. Such habitat variation is known to affect intraspecific variation in area used [32,33,34,35]. This is the first estimate for the Central Amazonia, which differs from previous study areas in topographic features, climatic conditions and vegetation composition, as well as being a highly productive environment where individuals may not need to roam far to meet their basic needs.

Although differences in the size of areas used by males and females is known for some terrestrial chelonians [36,37,34], we did not detect such sex-related differences. Previous studies indicated the rainy season, the period in which this study was carried out, as the mating season of *C*. *denticulatus*, which is the period when their home ranges are expected to be most dissimilar [1]. However, we did not detect individuals courting or copulating during the sampling period, and males and females were only observed moving together months after this study in late October and November. As tortoise behavior may shift seasonally and among areas, short-term studies, such as ours, may not be able to detect sex-related variation in behavior patterns [20,27].

The positive relationship between the body length and area used possibly reflects a higher demand for resources to meet metabolic needs [9]. Since tortoises grow continuously during their entire lives, larger individuals need longer displacements and larger areas to increase the probability of encountering essential resources, such as food, shelter and reproductive partner [38]. Conversely, are more sensitive to microclimatic variations, such as overheating [39], and are potentially more vulnerable to predation during dispersal movements [40]. While adult yellow-footed tortoises are mainly subject to predation by big cats [41], hatchlings and small tortoises are eaten by predators ranging from insects to a wide variety of mammals [8]. Size-related differences in habitat selection has rarely been demonstrated in terrestrial chelonians [36,34,42], because most studies have focused only on adult individuals [37].

With limited displacement, small *C*. *denticulatus* remained in small areas close or restricted to flat, elevated terrain, suggesting that these areas might reflect nest-site choice by females. Although information on reproduction of this species in the wild is lacking [43], several studies have shown that females of some tortoise species and other reptiles are highly selective in where they deposit their eggs [44,45,46]. It is suggested that yellow-footed-tortoise females often lay their eggs on the ground covered by a thin layer of leaves rather than burying them. This behavior was observed initially in captivity [47] and later observed in the field [8]. Egg laying on inclined areas could result in egg displacement by disturbances, such as rainfall, and compromise hatching success.

Higher flat areas had greater densities of fallen flowers, which are important components of yellow-footed tortoise diet [10] and especially suitable for hatchlings due to their tenderness. In addition, hatchlings and small tortoises need calcium for carapace growth [48], and the clay soils present in higher areas have been reported as being richer and more able to retain nutrients than the sandy soils from the lower areas [11,49]. However, Costa et al. al. [50] did not find a relationship between soil calcium and altitude in our study area. In contrast, adult individuals are able to move among areas in different elevations and access resources from a variety of topographic conditions.

We had expected to find a greater concentration of fruit on the ground in high elevations, as we did for flowers, but this was not so. Flowering usually occurs from June to October and fruiting from September to May in Duke Reserve [31], but changes in local climatic variables may influence the abundance of such resources. The El Niño effects in 2016, were the most severe that have been reported in the last 20 years [51], so our study may have missed the main fruiting season. We found lower fungus densities in the areas used by the tortoises than in other parts of the study site, but this is unlikely to reflect a causal relationship because fungus has been reported to be a significant fraction of the food items eaten by yellow-footed tortoises [10]. As the highest fungal densities recorded were on fallen trunks and the tortoises we followed usually deviated around these obstacles, the lower presence of fungus in the tortoise paths may be because the tortoises were avoiding trunks rather than fungi.

The lack of influence of food resources on the direction of displacement of *C*. *denticulatus* in Ducke Reserve may be a result of the high productivity of the rainforest, combined with the low energy demand and a diverse diet of yellow-footed tortoises [10]. Displacements of yellow-footed tortoises monitored on the Maracá Island, Northern Amazonia [8], also did not appear to be related to food, although that study did not evaluate the effects of food resources on hatchlings.

Although the yellow-footed tortoises we monitored did not select paths with more tree-fall gaps than those available in the environment, this is likely to be because of the high tree-fall abundance in the area. Fallen trees were clearly important resources for the individuals because the majority of large individuals were under tree-falls when encountered [18,9,8,20]. Shelters provide terrestrial chelonians with protection against predators and access to moderate temperatures and more humid environments [52,53,54]. While tangled branches around fallen trees allow large individuals to remain hidden with minimal metabolic rates, the open canopy nearby with direct sunlight makes fallen trees a potential thermoregulatory resource [8,18]. Previous studies have also reported that tree-fall gaps are an important resource for *C*. *denticulatus* [18,10,20], and are often used as shelter by tortoises [8,18]. In the Pinkaiti Reserve, Southeastern Amazonia, 30% of the *C*. *denticulatus* individuals were encountered in forest gaps larger than 25 m^2^ [29]. However, fallen trees are mainly important for larger individuals, and the majority of small individuals were found under the leaf litter or palm roots.

The thread-bobbins provided detailed information about space usage for both small and adult individuals. However, tortoises were sometimes lost because of line breakages, apparently caused by other animals crossing the area, which resulted in variation in monitoring period among individuals and may have caused some distortions in our estimatives of the daily distance covered by the yellow-footed tortoise. We also lost several individuals at the beginning of the study before we learned how to best attach the bobbins. Daily location of monitored individuals was often unachievable due to logistical limitations since simultaneous monitoring of various individuals would require large teams working throughout the study area, which should be considered in future studies. Additionally, although it is a method that has been applied successfully in other Amazonian areas [10], catch by pitfall traps was less efficient than active search in this study. Possibly, the rolling terrain and the heavy daily rains hindered the dispersal of bait odor over large areas. One additional difficulty was to determine the sex of small (young) individuals of the yellow-footed tortoise. Although there are some methods available (e.g. genetic analyses) or potentially useful (e.g. geometric morphometry) [55] for sex determination in some chelonians species, the use of such techniques was not possible in our study. For instance, the sex in *C*. *denticulatus* is likely determined by environment temperature rather than genetic differences [56], and there is no validation on geometric morphometry for this species. In conjunction, such methodological limitations may have impaired our ability to accurately access some behavioral characteristics of *C*. *denticulatus* in Ducke Reserve as well as sex-related differences in habitat use, especially among young individuals.

Our results indicate that variations in terrain slope and elevation affected space use by *C*. *denticulatus*, especially by small individuals. In addition, fallen trees are important resources for tortoises in Ducke Reserve, especially for larger individuals, though small individuals mainly hid in the litter. Small tortoises used smaller areas than larger tortoises, and occupied high flat areas, where fallen flowers were more available. *C*. *denticulatus* has a large distribution, covering most of Amazonia, and remains common in Ducke Reserve, though the population faces hunting pressure by urban dwellers on the perimeter of the Amazon´s largest city, but *C*. *denticulatus* is currently classified as Vulnerable under the International Union for conservation of Nature (IUCN) Red List [57] and Data Deficient under the Brazilian Red List [58]. Therefore, detailed information on its habitat requirements is necessary to predict areas of occurrence and support decision-making for habitat protection and reintroduction projects.
